# Oral microbiome diversity and mortality in metabolic syndrome: a cohort study

**DOI:** 10.1080/20002297.2026.2712017

**Published:** 2026-08-06

**Authors:** Lingfei Meng, Xiaohao Zhang, Xiaohua Zhuang, Wenpeng Cui

**Affiliations:** a Department of Nephrology, The Second Hospital of Jilin University, Changchun, Jilin Province, China; b Department of Cardiology, The Second Hospital of Jilin University, Changchun, China

**Keywords:** Oral microbial diversity, metabolic syndrome, all-cause mortality, NHANES, survival analysis

## Abstract

**Background:**

Metabolic syndrome (MetS) is associated with a higher risk of mortality. Oral microbiome diversity is related to health outcomes, but its role in forecasting MetS prognosis and the relevant pathogenic mechanisms is still largely unexplored.

**Methods:**

Data from 1,769 MetS patients were extracted from the National Health and Nutrition Examination Survey (NHANES) 2009–2012 cycle. To evaluate alpha diversity, the Shannon index, Faith's phylogenetic diversity (PD), observed operational taxonomic units (OTUs), and the Inverse Simpson index were determined. ALDEx2 was employed for differential abundance analysis in the high-diversity subgroup. Co-occurrence network analysis was subsequently conducted, followed by partitioning around medoids (PAM) clustering for community typing. Prognostic associations were validated through Cox regression.

**Results:**

Both the Shannon index (hazard ratio [HR] = 0.78, 95% confidence interval [CI]: 0.63–0.96) and the Inverse Simpson index (HR = 0.84, 95% CI: 0.74–0.96) were inversely associated with mortality. In the high-diversity subgroup, differential abundance analysis did not identify any OTU whose relative abundance differed significantly by survival status. According to network analysis, the deceased group exhibited a higher density of positive co-occurrences (283 vs. 224 edges) alongside a notable expansion of negative interactions (71 vs. 15 edges), which was a hallmark of declining community stability. Two clusters were identified through community typing, yet this classification was not significantly related to survival outcomes (CType_2 vs. CType_1: HR = 1.05, 95% CI: 0.73–1.49).

**Conclusions:**

Oral microbiome diversity exhibits a negative association with mortality among MetS patients. However, in the high-diversity subgroup, ecological network disruption, rather than alpha diversity or differential taxonomic richness, differentiates the deceased patients from the alive counterparts. This suggests that community structure is a promising risk marker with a higher sensitivity than diversity alone.

## Introduction

Metabolic syndrome (MetS) is diagnosed when an individual presents with three or more of five cardiometabolic traits below: hyperglycemia, hypertension, low high-density lipoprotein (HDL) cholesterol, central obesity or hypertriglyceridemia. A sedentary lifestyle and excess adiposity are widely recognized as the primary drivers of these conditions. MetS affects approximately 1/5–1/3 of global adults and doubles the cardiovascular disease risk [1]. It also leads to increased cardiovascular and all-cause mortality [2,3]. This mortality association is robust among individuals aged <65 years, but is insignificant among people aged ≥65 years, demonstrating its age-dependent prognostic significance [4]. MetS displays the typical features of long-term low-grade inflammation and impaired immune function, which thereby affects the oral microbiome. The oral cavity may therefore serve as a readily accessible site for observing MetS-mediated dysbiosis and the relevant prognostic implications [5,6].

The oral microbiota shows a high diversity, consisting of approximately 700 bacterial species that are mostly uncultivated, and they form a closely interrelated ecological system. The oral cavity of healthy subjects harbors a microbial community featured by high diversity and site specificity, and species related to dental caries and periodontitis are constantly being investigated [7]. Dysbiosis, a phenomenon of disrupted microbial homeostasis, may increase the risk of oral or systemic disorders. Oral presentations include caries, periodontitis, or even oral cancer, and systemic manifestations include endocrine, immune, and cardiovascular conditions [8]. The oral microbiota executes crucial physiological functions, such as digesting food, absorbing nutrients, and modulating metabolites. Therefore, for MetS patients, oral dysbiosis, which is associated with a shift to an inflammophilic microbial community and a loss of beneficial taxa, probably exacerbates systemic metabolic disorders and increases the cardiometabolic risk [9]. According to cross-sectional analysis, the oral microbiota composition is changed in MetS [10]. As suggested by differential clustering and oral microbial enrichment between MetS patients and normal subjects, the local oral microbiota is related to systemic metabolic diseases [11].

The diversity of the oral microbiome, in particular alpha diversity that indicates microbial evenness and richness, has been extensively identified as an indicator of a healthy microbiota. The reduced diversity is indicative of ecological disruption and related to unfavorable health outcomes for different people. Particularly, the declined oral microbiome alpha diversity, reduced probiotics, and increased pathogenic bacteria are associated with diabetes [12]. In recent NHANES-based studies, subjects with decreased oral microbiome alpha diversity had an increased mortality risk. Similarly, this association can also be detected in the general population and in patients with hypertension [13,14]. Yet studies that especially focus on MetS patients are lacking. It is therefore hypothesized that the decreased oral microbiome alpha diversity in MetS patients is related to an elevated all-cause mortality risk.

Using the National Health and Nutrition Examination Survey (NHANES)-derived data, this work focused on investigating the relationships of all-cause mortality with several oral microbiome alpha diversity indices, including the Shannon index, Faith’s phylogenetic diversity (PD), and observed operational taxonomic units (OTUs) in MetS adults, with adjustment for lifestyle, sociodemographic and clinical confounders. Illustrating this association contributes to non-invasive risk stratification and guides microbiome-targeted treatments, finally improving the health outcomes of these individuals.

## Materials and methods

### Study population

Baseline patient data were derived from NHANES, an ongoing cross-sectional survey that employs a nationwide, stratified multi-stage cluster sampling method. It systemically evaluates the health status, nutrition, and disease burden in the non-institutionalized US population through standard protocols, including in-home interviews, laboratory testing, and physical examinations [15]. The current study was carried out following the Strengthening the Reporting of Observational Studies in Epidemiology (STROBE) guidelines.

**Figure 1.**
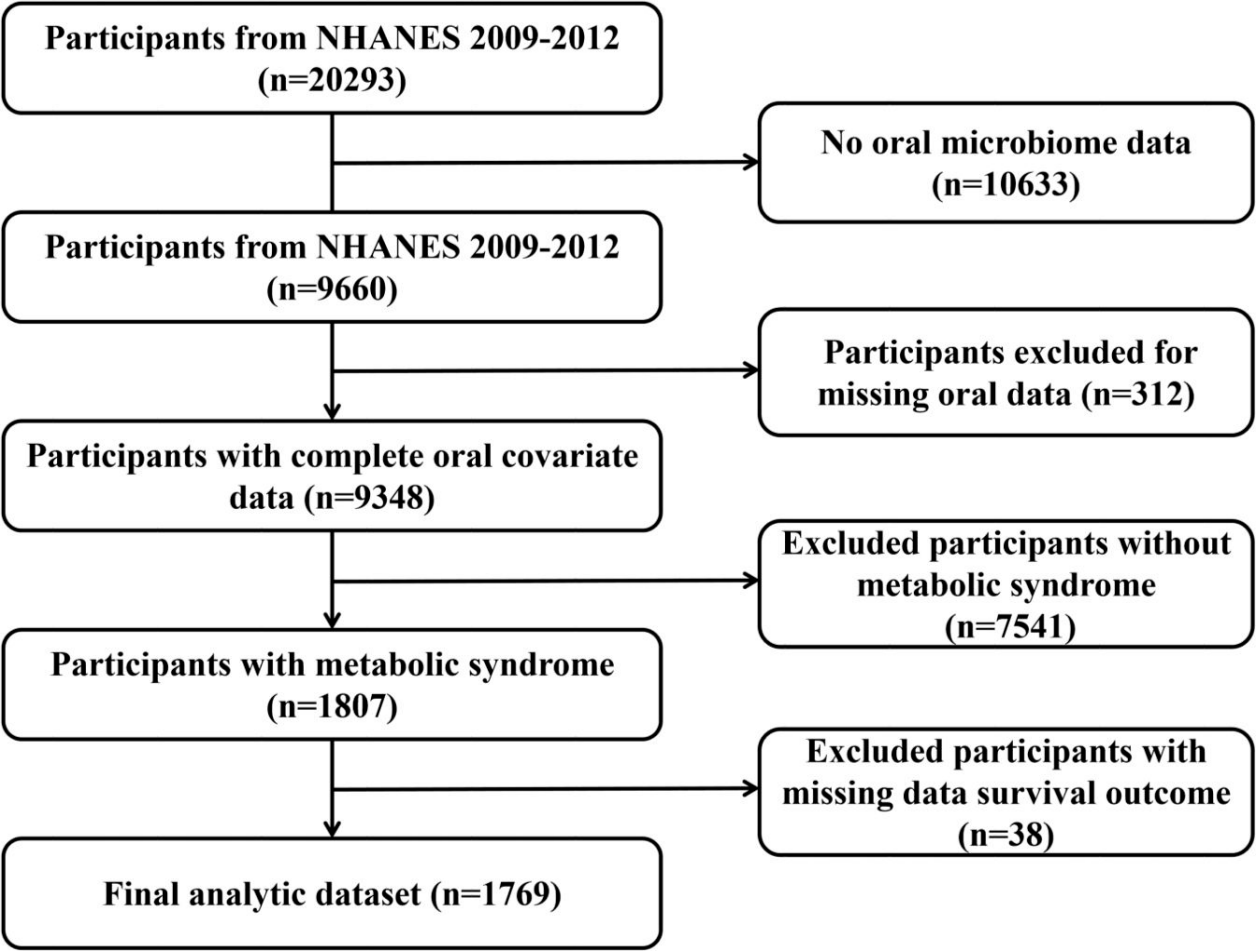
Participant recruitment and selection flowchart. Following the application of exclusion criteria, 1,769 participants with metabolic syndrome from the NHANES 2009–2012 cycle were included in the final analysis.

There were 20,293 subjects were recruited into the 2009–2012 cycles; among them, 9,660 were chosen, and their oral microbial samples with sequencing results were provided, whereas the remaining 10,633 were either ineligible or unselected, with unavailable microbiome data. Among the 9,660 subjects with available microbiome data, 312 were eliminated because of missing oral covariate data; finally, 9,348 subjects who had sufficient oral covariate data were obtained. Only subjects with MetS (*n* = 1,807) and adequate survival data (*n* = 1,769) were included, yielding 1,769 subjects for the final analysis (Figure 1).

### Oral microbiome diversity evaluation

Data regarding oral microbiome diversity were extracted from the NHANES Oral Microbiome Project carried out in subjects 14–69 years of age in the 2009–2012 cycles. One oral rinse specimen was obtained from every subject through swishing with a mouthwash solution. The Puregene DNA purification kit was then adopted for DNA extraction. Four alpha diversity indices were calculated on rarefied amplicon sequence variant (ASV) tables normalized to 10,000 reads per sample: the Shannon index, the Inverse Simpson index, Faith's PD, and observed OTUs. Of them, the Shannon and Inverse Simpson indices reflect both microbial richness and evenness, whereas Faith's PD and observed OTUs mainly capture richness alone. Beta diversity, which quantifies the compositional dissimilarity of microbial communities across individuals, was assessed using three metrics—unweighted UniFrac, weighted UniFrac, and Bray–Curtis dissimilarity—and was represented as a distance matrix [16]. These distance matrices were then visualized via principal coordinate analysis (PCoA). The first two principal coordinates were extracted and subsequently incorporated into weighted Cox regression models.

### Metabolic syndrome definition

The definition of MetS was made in line with the National Cholesterol Education Program (NCEP) Adult Treatment Plan III (ATP III) criteria [17] according to at least three components below:

– Increased waist circumference: ≥102 and ≥88 cm in male and female separately.
– Increased triglycerides: ≥150 mg/dL or taking drug medications.
– Decreased HDL cholesterol: <40 and <50 mg/dL in male and female separately, or taking drug medications.
– Increased blood pressure: systolic ≥130 mmHg, diastolic ≥85 mmHg, or undergoing antihypertensive treatments.
– Increased fasting glucose: ≥100 mg/dL or undergoing drug medications.

### Outcome ascertainment

All-cause mortality, which was evaluated based on the public NHANES Linked Mortality File, a program tracking survival status and death causes of subjects with unique respondent sequence numbers, was our primary endpoint. Follow-up data were collected up to December 31, 2019.

### Covariate assessment

Covariates were selected from previous studies that examined the relationship between oral health and mortality [13]. Structured questionnaires were adopted for collecting data on these covariates. Sociodemographic data were also extracted, including age, sex, race/ethnicity (Mexican American, other Hispanic, non-Hispanic White, non-Hispanic Black, other races), income (categorized as poverty income ratio [PIR] < 1, 1-3, or >3), and education level (grouped as lower than high school, high school graduate, or college graduate and above). Lifestyle-related covariates included smoking status (yes/no), alcohol consumption, body mass index (BMI, <25, 25–29.9, or ≥30  kg/m^2^) [18], physical activity (expressed as weekly total metabolic equivalents of task [METs] determined based on the Global Physical Activity Questionnaire) [14], and diet quality (evaluated by the Healthy Eating Index-2015 [HEI-2015]) [19]. The diagnosis of periodontitis was made according to loss of attachment (LOA) and probing pocket depth (PPD) based on the NHANES oral health examination, in strict accordance with the American Academy of Periodontology case definitions [20]. All subjects were divided into none/mild, moderate, or severe periodontitis groups by appropriate thresholds. Periodontitis was categorized as present (moderate/severe) or absent (none/mild) in the analysis. The identification of coronary heart disease was performed according to self-reported data from the subjects.

### Statistical analysis

Baseline subject features were reported based on survival status. Continuous data were represented by mean ± standard deviation (SD) and were analyzed by ANOVA, whereas categorical data were represented by counts and percentages and examined through the Rao–Scott chi-square test.

Multiple imputation by chained equations (MICE) was adopted for the management of missing covariate data. Five imputed datasets were obtained, with pooling of estimates by Rubin's rules. Covariates whose missing rates exceeded 10% were our priorities for imputation, whereas covariates that had smaller missing rates were first imputed with the median or mode (for continuous and categorical data separately), as reported in NHANES-based oral microbiome studies [19]. The missing observation number and percentage of every covariate are presented in Supplementary Table S3.

To appropriately account for the complex, multistage, clustered probability sampling structure inherent to the NHANES, Mobile Examination Center (MEC) examination weights were incorporated into every analytical procedure according to the recommendations of the Centers for Disease Control and Prevention (CDC) [21]. By utilizing the threshold of median, indices of oral microbiome alpha diversity were divided into high- or low-diversity subgroups for result visualization. Survival probabilities between these two subgroups were illustrated through the construction of Kaplan–Meier curves, and the two subgroups were compared by log-rank test. To analyze the association of oral microbiome alpha diversity with all-cause mortality, we utilized weighted Cox proportional hazards regression models that incorporated NHANES survey weights. Each oral microbiome alpha diversity index was modeled as a continuous variable. All Cox models were adjusted for the following covariates: age, sex, ethnicity, education, smoking status, BMI, alcohol consumption, periodontitis status, PIR, physical activity (METs/week), HEI-2015 score and coronary heart disease. For beta diversity, principal coordinates derived from Bray–Curtis, weighted UniFrac, and unweighted UniFrac distance matrices were similarly entered into weighted Cox regression models. Prior to these analyses, permutational multivariate analysis of variance (PERMANOVA; 999 permutations, implemented via adonis2) was used to assess overall compositional differences in the oral microbiome between mortality groups in both unadjusted and adjusted models (age, sex, race/ethnicity, BMI, smoking; by = ‘terms’).

Given the modest number of deaths (*n* = 132), analyses stratified by periodontitis status were performed with caution to avoid overinterpretation of potentially unstable effect estimates. Besides, formal interaction terms (alpha diversity × periodontitis) were included in the fully adjusted models to assess effect modification. The NHANES sampling weights were incorporated in all analyses.

### Differential abundance analysis

To identify specific taxa associated with mortality in the high-diversity subgroup, differential abundance analysis was carried out using ALDEx2, a compositionally-aware approach that explicitly accounts for the biases inherent to microbiome sequencing data. This method uses a centered log-ratio (CLR) transformation coupled with Monte Carlo sampling to estimate between-group differences in taxon abundance. Typically, taxa with an effect size >1 and a Benjamini‒Hochberg adjusted *P*-value < 0.05 were considered significantly abundant.

### Co-occurrence network analysis

To characterize ecological interactions among oral microbial taxa in the high-diversity subgroup, co-occurrence networks were constructed based on Spearman’s rank correlations calculated from genus-level relative abundances. To retain only associations with ecological relevance, we restricted the analysis to genera that were present in over 20% of the samples and had a mean relative abundance exceeding 0.001. Networks were constructed separately for the living and deceased groups, with positive edges (Spearman’s *r* > 0.3, *P* < 0.05) and negative edges (Spearman’s *r* < −0.3, *P* < 0.05) included. Network topological properties – including node count, edge count, density, modularity, average degree, and transitivity – were computed using the igraph package. To identify the most central taxa, genera were ranked by degree centrality, and the top 10 were designated as hub taxa.

### Community typing and prognostic validation

To identify microbial community types that operate independently of alpha diversity, partitioning around medoids (PAM) clustering was performed on the Bray‒Curtis distance matrix derived from genus-level relative abundances across all subjects. The optimal cluster number (k = 2) was determined by maximizing the average silhouette width over a range of k values from 2 to 6. The resulting community type labels were then entered as independent variables into weighted Cox proportional hazards models, adjusted for age, sex, BMI, and smoking, to evaluate their prognostic value. These models were run both in the full cohort and within the high-diversity subgroup (defined by the median Shannon index).

All statistical analyses were conducted using R software (version 4.4.1). All tests were two-sided, and a *P*-value below 0.05 was considered statistically significant.

## Results

### Baseline characteristics

Table 1 presents the baseline characteristics of the 1,769 subjects with MetS, stratified by survival status. Deceased subjects were significantly older than survivors [median (interquartile range, IQR): 60 (51, 65) vs. 49 (38, 60) years, *P* < 0.001]. Among the 132 deaths, which represented 7.46% of the total cohort, several risk factors were more prevalent than in survivors: smoking (71.2% vs. 46.7%, *P* < 0.001), periodontitis (78.0% vs. 57.5%, *P* < 0.001) and coronary heart disease (11.4% vs. 3.2%, *P* < 0.001). In addition, deceased subjects had a lower PIR: the proportion of individuals with a PIR below 1.0 was higher in the deceased group than in the alive group (29.5% vs. 24.1%), whereas the proportion with a PIR above 3.0 was lower among decedents (18.9% vs. 29.6%, *P* *=* 0.030). Education level also differed significantly between the two groups (*P* *=* 0.024), with a greater proportion of high school graduates in the deceased group (25.0% vs. 15.8%). No significant between-group differences were observed in sex, race/ethnicity, alcohol consumption, BMI, HEI-2015 score, or physical activity level (all *P* > 0.05). With respect to the alpha diversity indices, all four metrics were significantly lower in the decedents than in the survivors: observed OTUs (116.44 vs. 130.82, *P* < 0.001), Faith's PD (13.63 vs. 14.52, *P* = 0.006), the Shannon index (4.39 vs. 4.62, *P* *<* 0.001), and Inverse Simpson index (0.89 vs. 0.90, *P* = 0.040). Collectively, these findings suggest that oral microbial diversity is inversely associated with mortality risk in the MetS population.

**Table 1.** Baseline characteristics and alpha diversity by survival status among Metabolic Syndrome adults: NHANES 2009–2012 (n=1769).

Index	Survival (*n* = 1637)	Death (*n* = 132)	*p*
Age	49 (38, 60)	60 (51, 65)	<0.001
Gender (Male)	792 (48.4)	76 (57.6)	0.052
Race			
Mexican American	368 (22.5)	21 (15.9)	0.223
Other Hispanic	196 (12.0)	15 (11.4)	
Non-Hispanic White	637 (38.9)	62 (47.0)	
Non-Hispanic Black	321 (19.6)	28 (21.2)	
Other Race	115 (7.0)	6 (4.5)	
Education			
Under high school	212 (13.0)	16 (12.1)	0.024
High School	259 (15.8)	33 (25.0)	
College graduate	1164 (71.2)	83 (62.9)	
Alcohol Consumption	1209 (73.9)	105 (79.5)	0.182
Smoking	764 (46.7)	94 (71.2)	<0.001
BMI (kg/m2)			
<25	75 (4.6)	11 (8.3)	0.139
25−29.9	432 (26.4)	31 (23.5)	
≥30	1130 (69.0)	90 (68.2)	
PIR			
PIR < 1	394 (24.1)	39 (29.5)	0.030
1 ≤ PIR ≤ 3	759 (46.4)	68 (51.5)	
PIR > 3	484 (29.6)	25 (18.9)	
Coronary heart disease	53 (3.2)	15 (11.4)	<0.001
Periodontitis	941 (57.5)	103 (78.0)	<0.001
HEI-2015 score	53.02 (12.78)	51.84 (11.58)	0.304
Physical activity (METs/week)	3579.36 (10474.34)	2012.88 (4604.95)	0.088
Observed OTUs	130.82 (44.81)	116.44 (48.29)	<0.001
FaithPD	14.52 (3.52)	13.63 (3.80)	0.006
Shannon	4.62 (0.69)	4.39 (0.77)	<0.001
Inverse Simpson	0.90 (0.06)	0.89 (0.07)	0.040

BMI: Body Mass Index; PIR: Poverty Income Ratio.

### Associations between oral microbiome diversity and all-cause mortality

Over the course of follow-up, 132 deaths were documented. Among these, malignant neoplasms (31.1%) and heart diseases (25.8%) were the leading causes, jointly accounting for more than half of all fatalities. The additional causes were diabetes (6.1%), chronic lower respiratory conditions (5.3%), cerebrovascular disorders (4.5%), influenza/pneumonia (3.8%), accidents (2.3%), nephritis (0.8%) and other/unspecified causes (20.3%).

**Figure 2.**
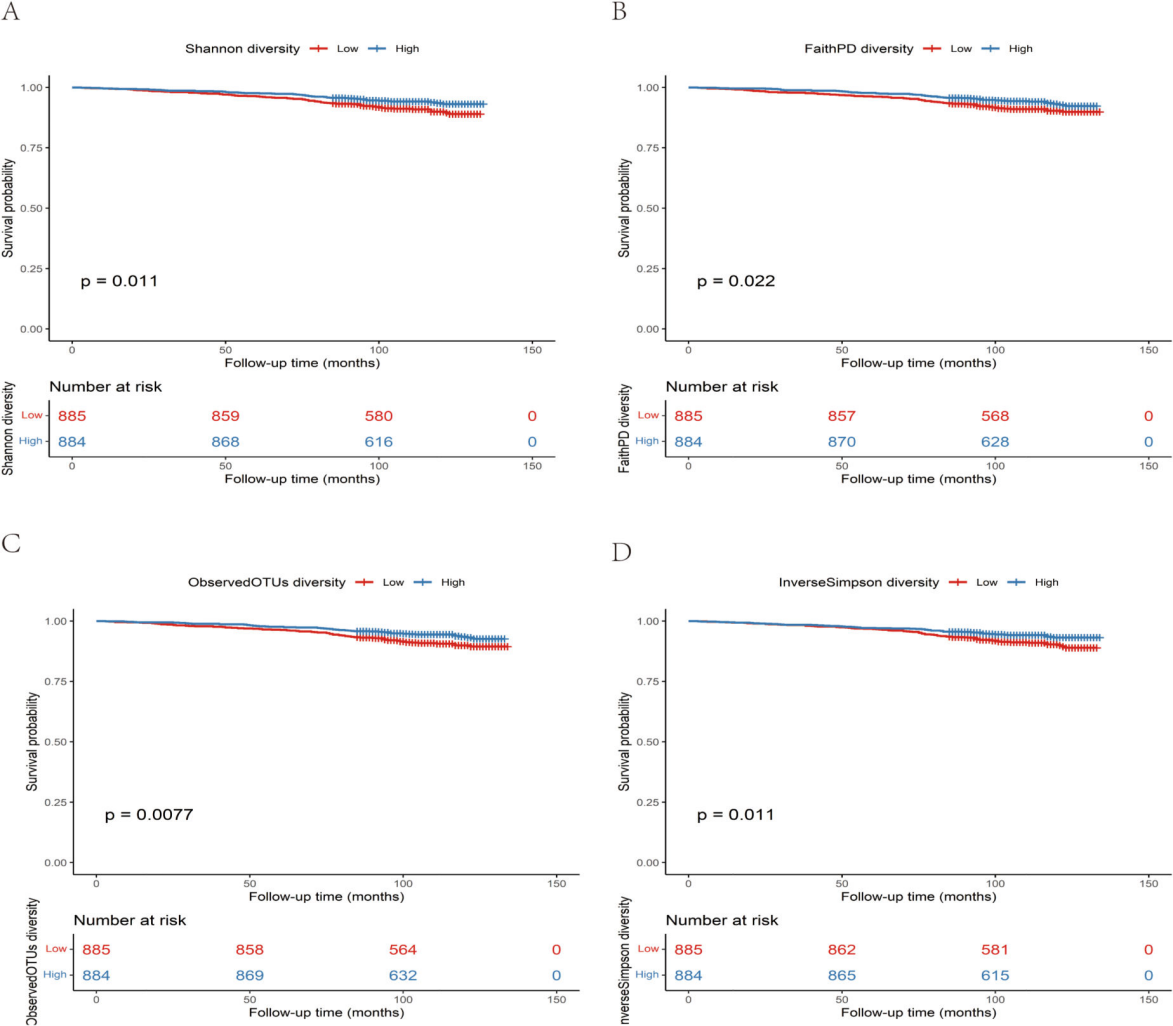
Kaplan‒Meier survival curves for all-cause mortality according to alpha diversity quartiles. (A) Shannon index, (B) Faith's phylogenetic diversity, (C) observed OTUs, and (D) Inverse Simpson index. Log-rank tests were used to derive the corresponding *P*-values.

Kaplan–Meier curve analysis was performed to compare survival between subgroups. As shown in Figure 2, the high-diversity subgroup exhibited markedly superior survival probabilities for each alpha diversity index, with significant differences according to the log-rank test (*P* < 0.05).

Associations were analyzed by weighted Cox proportional hazards models, with progressive adjustment for covariates (Table 2). In Model 4 (fully adjusted), a per-unit elevation of the Inverse Simpson index decreased the mortality risk by 16% (hazard ratio [HR] = 0.84, 95% confidence interval [CI]: 0.74–0.96, *P* *=* 0.013). This negative association was also detected for the Shannon index (HR = 0.78, 95% CI: 0.63–0.96, *P* *=* 0.018). Faith’s PD and observed OTUs did not show such associations in Model 4 (*P* > 0.05). From the above findings, richness- and evenness-related diversity indices are more closely related to survival in MetS individuals.

**Table 2.** Independent associations of oral microbiome diversity with all-cause mortality.

Index	Model 1		Model 2		Model 3		Model 4	
	HR(95% CI)	P	HR(95% CI)	P	HR(95% CI)	P	HR(95% CI)	P
FaithPD	0.83 (0.66–1.04)	0.110	0.97 (0.78–1.22)	0.822	1.01 (0.81–1.27)	0.912	0.93 (0.74–1.17)	0.524
Inverse Simpson	0.87 (0.77–0.98)	0.024	0.87 (0.76–1)	0.052	0.86 (0.74–0.98)	0.028	0.84 (0.74–0.96)	0.013
Observed OTUs	0.74 (0.56–0.97)	0.032	0.89 (0.68–1.17)	0.397	0.94 (0.71–1.23)	0.626	0.85 (0.64–1.12)	0.251
Shannon	0.75 (0.62–0.89)	0.002	0.8 (0.66–0.98)	0.034	0.82 (0.66–1.02)	0.078	0.78 (0.63–0.96)	0.018

For the analysis of oral microbiome diversity, we fitted four sequential Cox proportional hazards models with progressively adjusted covariates. Model 1 was unadjusted. Model 2 was adjusted for age, sex, race/ethnicity, income-to-poverty ratio, and educational level. Model 3 additionally adjusted for smoking status, alcohol consumption, body mass index, physical activity (METs/week), and diet quality (HEI-2015). Model 4 further adjusted for periodontitis status (presence/absence) and coronary heart disease. All models accounted for the complex survey design of NHANES.

**Figure 3.**
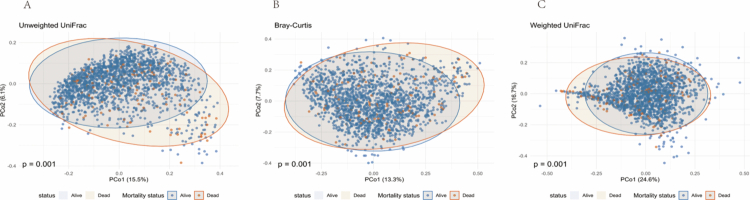
Principal coordinate analysis (PCoA) plots showing mortality status-stratified beta diversity. (A) Unweighted UniFrac, (B) Bray‒Curtis dissimilarity, and (C) weighted UniFrac. One point stands for one participant. Different colors indicate different survival statuses (alive vs. dead). PERMANOVA-derived *P*-values are shown.

PCoA was conducted on the basis of weighted UniFrac, unweighted UniFrac, and Bray–Curtis distances, revealing that the deceased group was modestly separated from the living group (Figure 3). As shown by unadjusted PERMANOVA using 999 permutations, these three distance matrices were significantly different between the two groups (*P* *<* 0.001). These associations were still significant when age, sex, race/ethnicity, BMI, and smoking status were adjusted (adjusted *P* *<* 0.05). To analyze the mortality-related microbial gradients, distance matrix-derived principal coordinates were extracted and incorporated as predictors into weighted Cox models separately, after sociodemographic variables, comorbidities, and lifestyle factors were adjusted progressively (Models 1–4). In Model 4, only the PCo2 of Bray–Curtis dissimilarity was closely related to all-cause mortality (HR = 1.29, 95% CI: 1.03–1.62, *P* = 0.026). The remaining principal coordinates were not statistically significant (*P* > 0.05).

### Stratified analysis and association of alpha diversity with periodontitis

Stratified analysis and formal interaction tests were subsequently performed to analyze whether the relationship between alpha diversity and mortality was affected by periodontitis. A high Shannon index in the non-periodontitis subgroup was strongly related to a decreased mortality risk (HR = 0.69, 95% CI: 0.48–0.99, *P* = 0.043). Additional alpha diversity indices were not significant in both strata (*P* > 0.05) (Table S1). As verified by interaction tests, periodontitis did not exert any obvious modification effect on all diversity metrics (*P* for interaction > 0.05) (Table S2). From the above observations, for non-periodontitis people, Shannon diversity may have a potential protective effect, but no definite conclusions can be drawn since formal interaction is lacking.

### Differential abundance in the high-diversity subgroup

ALDEx2 was employed for compositionally-aware differential abundance analysis to explore whether specific taxa can differentiate between living and deceased subjects in the high-diversity subgroup. There were no taxa meeting the significance criteria (effect size > 1, adjusted *P* < 0.05). Wilcoxon rank-sum tests were conducted in sensitivity analysis after correction for the false discovery rate (FDR), and 669 differential taxa were identified (BH-adjusted *P* < 0.05), yet the broad signal indicates the diffuse and taxonomically universal difference between the two subgroups, which is not caused by several keystone taxa (Figure S1).

### Co-occurrence network disruptions in deceased subjects of the high-diversity subgroup

**Figure 4.**
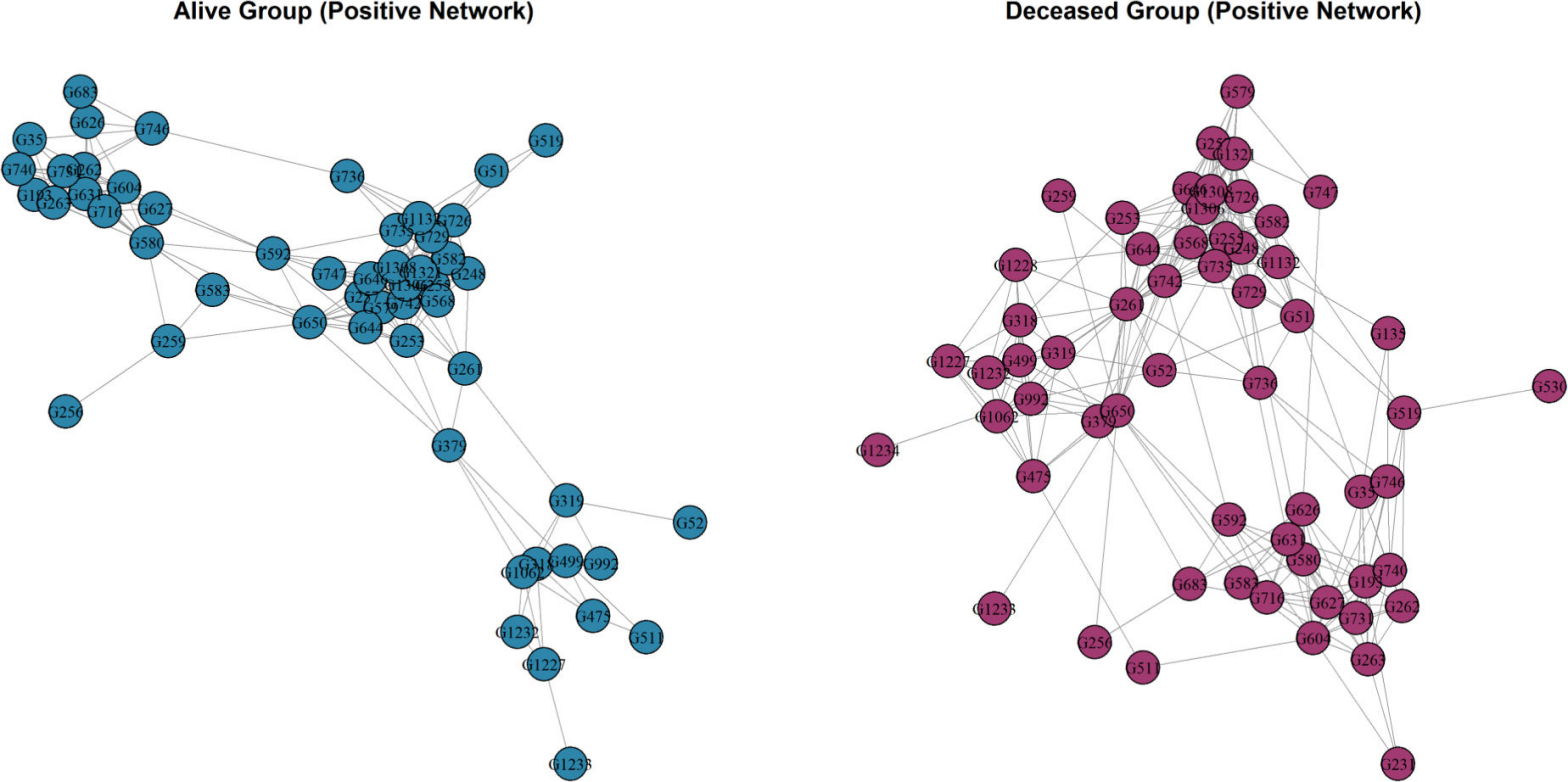
Side-by-side comparison of positive co-occurrence networks in the alive versus deceased groups.

Alpha diversity showed no significant difference between deceased and living subjects in the high-diversity subgroup, but differences in the ecological network structures were significant. Deceased subjects likely had closely connected positive connections (nodes: 58 vs. 53; edges: 283 vs. 224; average degree: 9.76 vs. 8.45) as well as markedly more negative edges (71 vs. 15). Moreover, an increased modularity (0.52 vs. 0.48) was detected in the deceased network, indicating that the community structure was fragmented regardless of greater edge density. This pattern – closely connected positive networks along with more negative edges and greater modularity – suggests that there is a community exposed to ecological stress, in which potent competitive interactions probably lead to isolated module formation (Figure 4).

### Community typing is not independently related to mortality

PAM clustering was conducted, which detected two optimal community types among these subjects (Figure S2), with CType_1 dominated by low diversity (92.0%), whereas CType_2 dominated by high diversity (73.7%). In Model 4, CType_2 did not show a close relation with mortality relative to CType_1 (HR = 1.05, 95% CI: 0.73–1.49, *P* = 0.798). CType_2 exhibited an increased risk in the high-diversity subgroup (HR = 3.07, 95% CI: 0.42-22.18), yet with no statistical significance (*P* = 0.267), probably because CType_1 had a modest sample size in the subgroup (*n* = 52) (Figure S3). According to these results, composition-based community typing alone is inferior to alpha diversity in predicting mortality, and network-level metrics are needed.

## Discussion

Oral microbial alpha diversity was first found to be inversely related to mortality risk in our nationally representative MetS subjects. The per unit elevation of the Shannon index reduced the mortality risk by 22%, while the per unit elevation of the Inverse Simpson index decreased the risk by 16%. The more potent relationship probably indicates that MetS individuals are metabolically vulnerable and usually present with long-term low-grade inflammation, endothelial dysfunction, and insulin resistance.

### Network disruption is a risk marker beyond alpha diversity

An important new finding in the present work is the fundamental differences in the ecological networks between the deceased and living subjects in the high-diversity subgroup, even though their Shannon indices were similar (4.64 vs. 4.62, *P* = 0.52). For deceased subjects, densely interconnected positive connections and markedly more negative edges were detected, indicating increased community stress and ecological destabilization. Consistently, an increased modularity (0.52 vs. 0.48) was also observed in the deceased network, suggesting an apparently more fragmented modular structure. Yet the higher modularity was accompanied by the markedly more negative edges (71 vs. 15). This finding demonstrates that strong competitive interactions across modules probably strengthen ecosystem resilience and stability, even though the network is fragmented into modules. Such network-level differences, which cannot be detected simply by alpha diversity, underscore the additional insights offered by network analysis. These results can explain why Adam et al. opinion regarding the coexistence of high diversity with periodontitis and mortality: rather than diversity itself, the ecological structure is the factor determining the protective or pathological nature of a community [22].

For the deceased subjects enrolled in this study, their sociodemographic variables and behavioral factors are consistent with the known social and behavioral factors determining health. Relative to living subjects, deceased participants were probably older, were smokers (71.2% vs. 46.7%), developed periodontitis (78.0% vs. 57.5%), and had a low PIR < 1.0 (29.5% vs. 24.1%). Such results align with previous studies reporting that smoking and old age decrease oral microbial diversity [23]. Periodontitis resulting from disturbance of the oral microbiota, may induce immune dysregulation, and these two factors are interrelated [24,25]. The low socioeconomic status is associated with poor oral health [26], while periodontitis and socioeconomic disadvantage are independently related to mortality. When the above possible confounders were adjusted in the multivariable models, the relationship of alpha diversity with survival was still potent, suggesting that oral microbiome diversity can independently predict the prognosis of MetS individuals.

### Alignment with earlier results and critical insights

These results align with several NHANES studies. For general US adults, an increased Shannon index is associated with a decreased mortality risk by 15% per standard deviation elevation [27], whereas contrasting extreme tertiles of ASV richness are related to a 37% lower risk [19]. Likewise, Mondal et al. discovered that OTU richness was negatively related to mortality [14]. The effect sizes detected in our subjects were greater (approximately 22 and 16% for the Shannon and Inverse Simpson, respectively), indicating the increased vulnerability in people with metabolic impairment.

This association is supported by other studies. According to Shen et al., a decreased mortality risk was independently associated with increased oral microbiome alpha diversity and superior diet quality; the hazard ratio (HR) for all-cause mortality in subjects within the highest tertiles for two factors was 0.37 (95% CI: 0.23–0.60) [19]. Yang et al. conducted an independent NHANES-based study on 8,224 adults and discovered that a decreased all-cause mortality risk was associated with increased oral microbiome diversity. The association could also be detected in subjects with diabetes, hypertension, or additional chronic diseases, suggesting that oral microbial diversity can be used to predict high-risk individuals [28]. Such protective associations are detected in multiple high‑risk populations, such as hypertensive people, and are still robust even when periodontitis status is accounted for [13,27]. This finding reveals that oral microbial diversity may exert its survival benefit via pathways that extend beyond periodontal health.

### Exploratory analysis on the association of alpha diversity with periodontitis

In accordance with earlier studies associating periodontitis with increased mortality [29], the baseline data in the present work demonstrated the markedly different periodontitis prevalence rates between the deceased (78.0%) and living (57.5%) groups. Severe periodontitis can lead to a 3-fold increase in cardiorenal mortality among diabetics relative to those with no severe periodontitis [30]. From our exploratory analysis, the beneficial value of alpha diversity remained largely unaffected by periodontitis status, which is in agreement with Mondal et al.'s mediation analysis reporting no obvious mediation by periodontitis or additional conventional risk factors [14]. The finding shows two key implications. First, the beneficial effect of oral microbiota diversity is probably achieved through mechanisms beyond periodontitis, such as systemic inflammation regulation, immunomodulation, and the nitrate–nitrite–nitric oxide (NO) pathway [31]. Through excess free fatty acids, abnormal adipokine production, and cholesterol-mediated NLRP3 inflammasome activation, metabolic dysfunction is increasingly suggested to change lipid mediator profiles to the pro-inflammatory state with persistent systemic inflammation and lipid metabolic dysregulation, thereby reinforcing the pro-inflammatory microenvironment and forming a vicious cycle associated with oral dysbiosis and systemic metabolic dysfunction [32]. Second, the maintenance of high microbial diversity shows protective effects even for those who develop periodontitis. This underscores that it is clinically important to preserve oral microbial richness, even though periodontal diseases are diagnosed. However, such exploratory interpretations should be validated in well-designed studies.

From the above results, it is crucial to select suitable diversity metrics and interpret non-significant findings obtained from one individual index with caution. With respect to *β*-diversity, distance matrix-derived principal coordinates were tested in Model 4. Only the PCo2 of Bray–Curtis dissimilarity was statistically significant in this model. Nevertheless, this result is exploratory owing to the small event count and the multiplicity of axes tested, and thus requires cautious interpretation.

### Possible mechanisms associating oral microbiota with mortality

#### Direct systemic effects of oral dysbiosis

After the loss of oral microbial diversity, pathogenic taxa will grow excessively, whereas beneficial commensals will be depleted, which can disrupt the oral ecological homeostasis. This dysbiosis is related to many systemic disorders, such as diabetes, atherosclerosis, autoimmune conditions, and even cancers; besides, it is associated with poor recovery from surgical wounds and infections. Therefore, oral dysbiosis has been considered a cause of unfavorable health outcomes, such as death [9]. Obstructive sleep apnea-hypopnea syndrome (OSAHS) can significantly alter the oral microbiota and facilitate cardiovascular pathology via oxidative, inflammatory, and thrombotic pathways [33]. Oral dysbiosis, which especially involves *Porphyromonas gingivalis*, may result in cardiovascular disorders through multiple pathways: enhanced trimethylamine *N*-oxide (TMAO) secretion, direct vascular invasion, and systemic inflammation [31].

#### The nitrate–nitrite–NO pathway

NO, an important factor regulating vascular tone, is produced by dietary nitrate in two steps. Oral bacterial species first convert nitrate into nitrite, and subsequently reduce nitrite to NO. High-nitrate diets can enrich NO-producing taxa (such as Rothia and Neisseria) and suppress other taxa, resulting in higher NO bioavailability and lower blood pressure, in particular in the elderly [34]. This pathway is impaired by the loss of oral microbiota diversity among MetS individuals, which may exacerbate endothelial dysfunction and hypertension. As supported by population-based evidence, a greater nitrate-reducing oral bacterial abundance is associated with lower fasting glucose, systolic blood pressure, and insulin resistance [35]. Apart from nitrate reduction, nitrite metabolism seems to be crucial, and a microbial shift to ammonia (NH₃), but not NO, production is related to an unfavorable cardiometabolic profile, while the increased NO-to-NH₃ ratio indicates superior metabolic health [36]. Consequently, the loss of oral microbial alpha diversity among MetS individuals can impair NO bioavailability by diminishing nitrate reduction and impairing the nitrite scavenging capacity, which contributes to hypertension, systemic inflammation and insulin resistance, finally giving rise to the increased mortality risk detected in these individuals [36].

### Oral–gut axis

Oral dysbiosis may exert more extensive effects via the oral–gut axis [37,38]. Pathogenic oral bacterial species, including *P. gingivalis*, disrupts the gut microbial ecosystem, change metabolite generation of metabolites (such as SCFAs and TMAO), and impairs intestinal barrier function [39,40]. These disturbances thereby aggravate low-grade systemic inflammation and metabolic endotoxemia, while these conditions can further exacerbate obesity, dyslipidemia, and insulin resistance [41,42]. Therefore, oral dysbiosis can amplify the inflammatory and metabolic burden to indirectly elevate the mortality risk among MetS patients. Maintaining oral microbial stability contributes to host–microbe homeostasis, limits pathogen expansion and safeguards beneficial bacteria.

In addition to those aforementioned mechanisms, oral dysbiosis may influence systemic health via other pathways, such as microbial translocation (transport of oral bacteria to distant organs), superantigens (activation of non-specific T-cells), and molecular mimicry (cross-reactive immune reactions) [9]. Loss of oral microbial diversity is not just observed in MetS; instead, it is also detected among diverse conditions like conditions, such as Alzheimer’s disease [43], severe psoriasis and psoriatic arthritis [44], atopic dermatitis [45], oral squamous cell carcinoma [46], and primary Sjögren’s syndrome [47], and during the progression from gingivitis to periodontitis [48]. Based on the transdiagnostic pattern, reduced oral microbial diversity is a common ecological marker of unfavorable health outcomes, which reinforces the prognostic implications of oral microbiota diversity in MetS. However, the transdiagnostic pattern also suggests the lack of diagnostic specificity of these diversity measures for a single disease, such as MetS.

### Modifiable factors

The observed association is of translational promise, given that the oral microbiome remains accessible to therapeutic intervention. For instance, freeze-dried *Lactobacillus zeae* N165 demonstrates periodontal benefits through reducing alveolar bone resorption by approximately 49% while inhibiting the production of critical inflammatory factor production (TNF-*α* and IL-6). Besides, it can help preserve gut and oral microbial profiles similar to those of healthy subjects. From the mechanistic perspective, this strain probably acts via multiple mechanisms: restricting glutamine degradation to prevent ferroptosis, regulating the lipopolysaccharide contents, and influencing biotin metabolism and polyketide glycan unit biosynthesis [49]. But more studies are warranted to analyze the potential translation of these probiotic measures into additional diseases. It is possible to maintain or restore oral microbial diversity via multiple ways. Different from broad-spectrum antimicrobials that usually decrease overall diversity, targeted nanomedicine contributes to the selective elimination of keystone pathogens and the preservation of commensal communities [24]. Some probiotic strains (including *Lactobacillus reuteri*, *Lactobacillus rhamnosus*, and *Bifidobacterium* species) are beneficial for periodontal therapy, such as reducing inflammation and plaque [50]. However, owing to the lack of long-term data and the presence of study heterogeneity, it is impossible to make definitive conclusions, and well-designed clinical studies are warranted. Oral sodium nitrate can mitigate MetS phenotypes in mouse models through restoring the balance of macrophage polarization by the sialin pathway. In this regard, nitrate metabolism is probably associated with metabolic health via this pathway [51,52].

### Future research directions

Future studies focusing on certain translational and methodological directions must be conducted. At present, sequencing technologies are unable to differentiate between living and dead bacteria, and dead cells comprise almost 50% of salivary samples; thus, PMA pretreatment can more accurately assess the samples [53]. Through metagenomic sequencing and molecular pathological epidemiology, specific pathways can be identified, and individualized microbiome-targeted therapeutic strategies can be formulated [54]. Further research must prioritize longitudinal designs with repeated measures, alongside interventional approaches such as probiotic or dietary modifications. Moreover, in the absence of significantly different global diversity metrics, changes in certain taxa are valuable biomarkers [55].

### Limitations

Certain limitations should be noted in the present work. Given its observational nature, no causal relationship can be inferred. Subgroup analysis was limited by the low number of deaths (*n* = 132), probably resulting in inaccurate estimates – in particular for beta diversity metrics. The oral microbiome is unstable, but it always changes with medications, diet, or additional exposures. Single-timepoint evaluation thereby provides only a cross-sectional snapshot, which does not faithfully reflect long-term stability. In addition, only US adults were recruited for this work, probably limiting the generalizability of our findings to additional populations or settings. No individual‑level taxonomic composition data were available in this work, making it impossible to identify specific taxa related to mortality. Future investigations incorporating metagenomic sequencing are warranted to connect diversity measures to distinct bacterial signatures. Notably, salivary microbiota cannot comprehensively capture the diversity present in additional oral niches, such as the dorsal tongue or subgingival biofilm, each of which harbors a unique microbial community and may exhibit different relationships with systemic health. MetS individuals usually take multiple medications (including statins, metformin, and antihypertensives), and these medicines can probably affect the oral or gut microbiome. Polypharmacy can therefore independently change oral microbial diversity and potentially confound the detected associations. More details of medications are unavailable, which is acknowledged as a source of residual confounding and an important priority for subsequent research. Finally, although the covariates were comprehensively adjusted, there may still be a certain degree of residual confounding.

## Conclusion

Taken together, these findings suggest that oral microbiome alpha diversity is negatively related to all-cause mortality among MetS patients. According to the exploratory interaction analysis results, this relationship remains unaffected by periodontitis status. The above results expand the findings of previous NHANES-based studies to individuals with metabolic impairment, and underscore the role of oral microbial ecology in prognosis prediction. Yet they must be interpreted with caution, considering the low power.

## Supplementary Material

supplementary material

Supplementary_tables.docx

Supplementary Material Supplementary Material

Supplementary Material Supplementary Material
