# Supplementary material for: Oral microbiome diversity and mortality in metabolic syndrome: a cohort study
Source: J Oral Microbiol. 2026 Aug 6;18(1):2712017. doi: 10.1080/20002297.2026.2712017 (PMC13449441; doi:10.1080/20002297.2026.2712017)
Supplement: Supplementary file 1 [file ZJOM_A_2712017_SM3796.docx]

Table S1. Independent associations of oral microbiome diversity with all-cause mortality stratified by periodontitis status.

| Index | Periodontitis | | No periodontitis | |
| --- | --- | --- | --- | --- |
|  | HR(95% CI) | P | HR(95% CI) | P |
| Faith PD | 0.99 (0.74–1.32) | 0.947 | 0.74 (0.4–1.34) | 0.282 |
| Inverse Simpson | 0.87 (0.72–1.06) | 0.160 | 0.82 (0.62–1.07) | 0.127 |
| Observed OTUs | 0.93 (0.67–1.27) | 0.626 | 0.62 (0.33–1.19) | 0.138 |
| Shannon | 0.83 (0.63–1.11) | 0.211 | 0.69 (0.48–0.99) | 0.043 |

Table S2. Interaction of oral microbiome diversity and periodontitis status in relation to all-cause mortality.

| Index | HR(95% CI) | P |
| --- | --- | --- |
| Faith PD*Periodontitis | 1.14 (0.71–1.85) | 0.584 |
| Inverse Simpson*Periodontitis | 0.88 (0.67–1.16) | 0.365 |
| Observed OTUs*Periodontitis | 1.10 (0.64–1.90) | 0.730 |
| Shannon*Periodontitis | 1.01 (0.66–1.53) | 0.966 |

Table S3. Missing data patterns for covariates.

| Index | Missing (n) | Missing (%) |
| --- | --- | --- |
| Age | 0 | 0.0 |
| Gender (Male) | 0 | 0.0 |
| Race | 0 | 0.0 |
| Education | 34 | 1.9 |
| Alcohol Consumption | 113 | 6.4 |
| Smoking | 34 | 1.9 |
| BMI (kg/m²) | 8 | 0.5 |
| PIR | 134 | 7.6 |
| Coronary heart disease | 34 | 1.9 |
| Periodontitis | 423 | 23.9 |
| HEI-2015 score | 231 | 13.1 |
| Physical activity (METs/week) | 4 | 0.2 |
| Observed OTUs | 0 | 0.0 |
| Faith’s phylogenetic diversity | 0 | 0.0 |
| Shannon index | 0 | 0.0 |
| Inverse Simpson index | 0 | 0.0 |
